# Full Arch Implant-Prosthetic Rehabilitation in Patients with Cardiovascular Diseases: A 7-Year Follow-Up Prospective Single Cohort Study

**DOI:** 10.3390/jcm13040924

**Published:** 2024-02-06

**Authors:** Bianca D’Orto, Giulia Tetè, Matteo Nagni, Riccardo Federico Visconti, Elisabetta Polizzi, Enrico Felice Gherlone

**Affiliations:** 1 Dental School Department of Dentistry IRCCS San Raffaele Hospital, Vita-Salute San Raffaele University, 20132 Milan, Italy; tetegiulia92@gmail.com (G.T.); nagnimatteo@hotmail.it (M.N.); riccardo.visconti@hotmail.it (R.F.V.); gherlone.enrico@hsr.it (E.F.G.); 2 Chair Center for Oral Hygiene and Prevention, Dental School Department of Dentistry IRCCS San Raffaele Hospital, Vita-Salute San Raffaele University, 20132 Milan, Italy; polizzi.elisabetta@hsr.it

**Keywords:** dental prothesis, implant supported, dental implants, cardiovascular disease, systemic diseases

## Abstract

**Aim**: The rising average age increases edentulous cases, demanding more implant–prosthetic rehabilitation, with cardiovascular diseases being significant factors. This study compared healthy patients (CG = Control Group) and those with cardiovascular disease (TG = Test Group) for implant survival, Marginal Bone Loss (MBL), peri-implant tissue level parameters as Periodontal Screening and Recording (PSR), Plaque Index (PI), Bleeding on Probing (BoP) Peri-implant Probing Depth (PPD), and surgical complications. Smoking impact on both groups and medication influence in the TG were secondary outcomes. Patients underwent full-arch implant prosthetic rehabilitation. **Methods**: Implant survival rate, MBL, and surgical complications were recorded during the monitoring period (7 years), while peri-implant parameters were assessed at the end of the observational time. A total of 26 and 28 CG and TG patients were recruited, respectively. **Results**: A total of 128 implants were placed in CG, while 142 in the TG. Implant survival and MBL showed no significant differences (*p* > 0.05). Nevertheless, peri-implant parameters were more unfavorable in TG. The only significant surgical complication was higher bleeding rates in the TG (*p* < 0.05). **Conclusions**: Cardiovascular patients showed similar implant survival and MBL but had adverse peri-implant parameters and increased bleeding rates. Higher smoking levels may relate to unfavorable implant outcomes. Further investigation is needed on drug impact with larger samples.

## 1. Introduction

Edentulism is a significant issue that becomes more prevalent with the increase in the population’s average age. As people age, they become more susceptible to tooth loss due to various factors such as periodontal disease, dental decay, and wear and tear over time. The loss of teeth could impact a person’s oral health, overall well-being, and quality of life [1,2].

Dental implants supporting fixed prostheses could be considered the most requested rehabilitation by patients, to be provided with a therapeutic alternative that allows morphological and functional restoration, conferring aesthetic benefit and better psychological acceptance [3,4,5].

In totally edentulous patients, the absence of teeth leads to various changes in the surrounding bone and soft tissues over time. These changes are collectively referred to as “resorption” or “involution” of the maxillary bones. The lack of tooth roots and the absence of functional forces on the jawbone trigger a remodeling process that can result in the following conditions: reduction in residual bone height, superficialization of vasculo-nervous structures, and, in the upper jaw, pneumatization of the maxillary sinus [6,7,8].

Although, when possible, axial implants should be preferred [9], tilted implants supporting implant–prosthetic rehabilitation with the all-on-four method have provided good short- and long-term results [10,11,12], making it possible to avoid more invasive procedures such as bone grafting and sinus lift techniques [13,14].

In both cases of edentulism and the aging population, there is an increased incidence of systemic pathologies that can impact the placement and long-term survival of dental implants. These systemic pathologies could pose challenges and must be carefully considered during the treatment planning process. Some of the key systemic health factors that may affect dental implants include bone density and healing capacity, diabetes, immunosuppression, medications, smoking and tobacco use, and cardiovascular diseases [15,16].

Cardiovascular diseases (CVDs), which include hypertension, stroke, congenital heart disease, rhythm disorders, subclinical atherosclerosis, coronary artery disease, heart failure, valvular heart disease, and venous and peripheral diseases, affect approximately 12 million people each year, thus representing, together with diabetes, among the most widespread diseases [17,18].

In this regard, several authors have performed investigations into the impact of CDV and related drugs on dental implants.

Wang et al. [19] investigated the association between peri-implantitis and cardiovascular disease in 128 patients with dental implants. Although a higher prevalence of peri-implantitis was found in the cardiovascular disease group, controlling for confounders weakened the association.

The same author also explored pro-inflammatory cytokine profiles in patients with and without cardiovascular disease and peri-implantitis. The study revealed an increased expression of pro-inflammatory cytokines, supporting a weak association between chronic peri-implantitis and cardiovascular disease [20].

The study by Kaura et al. [21] evaluated the risk of hemorrhage in patients treated with single and double antiplatelets undergoing dental implants. The study concluded that dental implants can be placed safely in patients taking single antiplatelet drugs without discontinuing them. For patients treated with dual antiplatelets, the risk of bleeding was mild to moderate if the drugs were continued.

Dawoud’s systematic review focused on hemorrhagic complications in anticoagulated patients undergoing dental implantation. The review suggested that the continuation of anticoagulants in the peri-operative phase during dental implant surgery increases the risk of clinically insignificant peri- and postsurgical hemorrhage [22].

The aim of this study was to investigate and compare healthy patients (CG = Control Group) and patients with cardiovascular disease (TG = Test Group) concerning implant survival rate, marginal bone loss, peri-implant parameters, and surgical complications (first outcome). 

In addition, in each of the two groups the influence of smoking on previous values and, in patients with cardiovascular disease, the impact of medication (secondary outcomes) was assessed.

The null hypothesis was that there were no statistically significant differences between groups and that smoking and drugs had no statistically significant impact on the examined parameters.

## 2. Materials and Methods

This study followed a prospective longitudinal design. The research aimed to investigate healthy patients and patients with cardiovascular disease. The primary objective was to assess several critical factors related to dental implant procedures, including implant survival rate, marginal bone loss, peri-implant parameters, surgical complications, and the potential influence of smoking and medication use on these variables. 

Subsequently, a comparative analysis was conducted to investigate possible differences between the two groups concerning implant survival rate, marginal bone loss, peri-implant parameters, and surgical complications.

All procedures executed in this study involving human participants were in accordance with institutional and/or national research committee ethical standards and the 1964 Declaration of Helsinki and its subsequent amendments or comparable ethical standards. The ethics committee approval number was CE/INT/10/2015 (Ethic Committee Name: MPLANTO; Approval Code: 190/INT/2021; Approval date: 15 December 2021). 

The study was conducted at the Dentistry Department of the IRCCS San Raffaele, Milan, Italy.

Patients were enrolled from January 2016 to November 2016; the study data were collected from the date of patient recruitment until January 2023. 

### 2.1. Participants

All patients were provided with a medical anamnestic questionnaire that included interceptions of past and current systemic diseases and medications taken in the past and currently; they were also asked to include any smoking habits and the number of cigarettes smoked per day.

Subsequently, the dental evaluation consisting of objective examination and level I radiographic investigations (intra-oral radiographs and orthopantomography) was performed. 

The inclusion criteria were the following: age over eighteen years, absence of systemic diseases or presence of only cardiovascular disorders, edentulousness of one or both dental arches, severe impairment of the residual dentition with no possibility of recovering any of the dental units, and request for implant-prosthetic rehabilitation.

Patients having prospects of restoring one or more teeth, absolute contraindications to dental implant placement, i.e., bisphosphonates medication or radiotherapy of the head and neck performed in the last year, un-compensated cardiovascular diseases, presence of other systemic disorders, inability to adhere to protocol checks, and oral hygiene sessions and/or economic infeasibility in providing treatment were excluded.

According to the absence of any systemic diseases (CG) or the presence of cardiovascular diseases (TG), the sample was divided into two groups.

### 2.2. Implant-Prosthetic Protocol

#### 2.2.1. Pre-Surgical Protocol

All diagnoses were performed clinically and radiographically. A radiographic investigation was firstly made with panoramic radiography and secondly with cone beam computed tomography (CBCT) to assess the height and width of the residual bone. 

Blood tests were prescribed about one month before surgery to check the patient’s general health and to identify possible signs of cardiovascular diseases [23].

Concerning group B, the referring cardiologist was involved to assess that the cardiovascular condition was stabilized, and the patient was medically qualified for surgical treatment. INR for the warfarin group was checked 24 h before surgery and ranged between 1.87 and 3.19. Management recommendations for invasive dental treatment in patients on oral antithrombotic medication were followed: no patient had International Normalized Ratio (INR) ≥ 3.5 [24].

Antibiotic prophylaxis (2 g amoxicillin 1 h before surgery, 2 g clarithromycin in case of penicillin allergies) was only given to patients subjected to hypertension, cardiac pathologies, or association of both [25].

#### 2.2.2. Surgical Procedure

All procedures were performed according to safety protocols, relying on the period in which they were executed [26]. Surgery was performed under anesthesia induced by local infiltrations of optician 120 solutions with adrenaline 1:80,000 (AstraZeneca, Milan, Italy).

A crestal incision, made in the palatal or lingual direction to obtain more keratinized tissue, and medial and distal vertical release incisions provided a full-thickness flap. 

The exposed bone ridge was leveled with a straight handpiece and osseous forceps. The midline, maxillary sinus region, and mental nerve were defined with a sterile pencil as marker sites for fixtures’ positioning.

Depending on the height of the residual bone, as seen by CBCT [27], either six straight implants or two straight implants and two implants tilted mesially and distally were placed. A lanceolate drill was employed to drill the cortical bone. A ø 2.00 pilot drill was applied to provide a path for implant insertion and to define the fixture setting. A positioning pin was inserted to verify implant position, emergence, and, when occurring, angulation. Progressive diameter drills were applied to the final diameter of the fixture. The site was overprepared vertically and underprepared transversely to promote primary mechanical stability. The insertion torque varied between 30 and 40 N-cm before final implant placement, allowing for immediate loading. When implant insertion was incomplete, a manual screwdriver was employed. 

When six axial fixtures were placed, straight or 17-degree angled abutments were applied to compensate for any lack of parallelism.

In all-on-four protocol cases, 30- or 45-degree angled abutments were screwed on tilted implants and straight on axial ones.

Flap adaptation and suturing were performed around the closure screws or the extreme abutment with 3-0 resorbable sutures (Vicryl; Ethicon, Johnson & Johnson, New Brunswick, NJ, USA).

#### 2.2.3. Post-Surgical Protocol

Immediately after surgery, a panoramic radiography was performed to verify the correct implant position.

Antibiotic therapy (amoxicillin and clavulanic acid 1 g or clarithromycin 1 g in case of allergy, twice daily for 6 days after surgery) and analgesic therapy (non-steroidal anti-inflammatory drugs, as needed) were prescribed for each patient. Mouth rinsing with a chlorhexidine–digluconate-containing solution (0.20%) was recommended twice daily for 10 days. A vial of tranexamic acid was given to the patient in combination with sterile gauze to counteract any post-surgical bleeding [28,29,30].

#### 2.2.4. Prosthetic Protocol

Titanium cylinders were screwed on the abutments; a provisional all-acrylic resin prosthesis, obtained from preliminary impressions taken one week before surgery, was drilled at them to take pick-up impressions (Permadyne, ESPE, Seefeld, Germany).

Approximately 3 h after surgery, a screw-retained provisional denture was delivered, made of metal-reinforced acrylic, with a maximum of 12 teeth and no cantilever. The screw access holes were covered with temporary resin (Fermit, Ivoclar Vivadent, Naturno, Bolzano, Italy). Four months later, the provisional denture was replaced with an implant-supported permanent denture made of acrylic resin with a titanium framework and fitted with a distal cantilever [31,32].

Articulating paper (Bausch, Nashua, NH, USA), which reproduced the natural dentition, was applied to control the occlusion.

The screw access holes were covered with acrylic resin (Fermit, Ivoclar Vivadent Naturno, Bolzano, Italy).

#### 2.2.5. Follow-Up

Follow-up visits were performed 1 week after surgery, at 3 and 6 months, and then once a year for the next 7 years. Professional oral hygiene sessions were carried out every 4 months after the surgical–prosthetic procedure [33].

### 2.3. Clinical Outcomes

1. Comparison between CG and TG for the following criteria:
  - Implants Survival Rate
    The implant survival rate was based on implant loss during the follow-up period. After evaluating the implant survival rate in each group, CG and TG were compared to establish whether there were differences between healthy patients and patients with cardiovascular diseases.
  - Marginal Bone Loss
    Throughout each session, three blinded operators, all dentists, were actively engaged in the analysis of MBL values. The distribution of responsibilities among the operators was as follows: one conducted the radiographs, another handled the calibration process, and the third was responsible for data collection. Intra-oral X-rays were taken at 3, 6, and 12 months and once a year during the follow-up period. The software, (DIGORA 2.5, Soredex, Tuusula, Finland), was calibrated for each image using the known diameter of the fixture at the most coronal portion of the implant neck to assess marginal bone differences. The linear distance between the most coronal point of the bone-implant contacts and the coronal margin of the implant neck was measured on the mesial and distal sides, to the nearest 0.01 mm, and then the average was calculated. Bone level changes of individual implants were averaged at the patient level and then at the group level. Marginal bone loss has been compared between healthy patients and patients with cardiovascular diseases.
  - Peri-implant parameters
    The parameters were evaluated at the end of the follow-up period, i.e., 7 years after prosthetic loading. Two blinded operators were engaged in the process, with one clinically overseeing the measurements (dentist or dental hygienist), and the other managing the data collection. Periodontal screening and recording (PSR), plaque index (PI), bleeding index (BoP), and peri-implant probing depth (PPD) were recorded [34].
  - Surgical complications
    Possible clinical complications such as post-surgical edema, pain while taking analgesic drugs, bleeding, and/or wound infection were recorded during follow-up checks. Surgical complications have been compared between healthy and patients with cardiovascular diseases.

2. Impact of smoking on parameters recorded for the CG and TG

After comparing the two groups, the impact of smoking on implant survival rate, marginal bone loss, peri-implant parameters, and surgical complications was assessed in each.

Only smoking patients were considered, divided according to the number of cigarettes smoked per day (greater or less than ten per day), and the possible correlation with the above parameters was assessed.

3. Impact of drugs administered to patients with cardiovascular diseases

In the TG, the impact of drugs on implant survival rate, marginal bone loss, peri-implant parameters, and surgical complications was evaluated.

According to medical history, patients with cardiovascular disease were divided according to the medication category undertaken.

### 2.4. Statistical Analysis

Statistical analysis was carried out using Python 3.8.5 and the following packages: Math, SciPy. Stats, and Pandas. According to the sample distribution, variance, and experimental setting, we used parametric independent samples *t*-test, Pearson’s Chi-Square test, or z-test to test for/against differences between groups. To assess the evolution of implant survival and the risk factors over time, we have performed Kaplan–Meier and Cox hazard analysis. The hypothesis tests concerning the difference between groups were evaluated by considering patients as analysis units, while the survival analysis and the hypothesis test concerning implant failure considered implants as analysis units. 

Across all analyses *p*-values < 0.05 were considered significant. The statistical examination was conducted at a 95% significance level. 

The determination of the study size was guided by a statistical power analysis, employing a *t*-test for two independent samples. We aimed for a significance level (α) of 0.05 and a power (1-β) of 0.80, representing a commonly accepted balance between Type I and Type II errors. We calculated the required sample size for a two-sample *t*-test using the formula:

*n* = 2(σ^2^) (Z_α/2_ + Z_β_)^2^/δ^2^

where σ is the estimated standard deviation, Z_α/2_ and Z_β_ are critical values for the chosen significance level and power, and delta is the effect size.

We conducted a sensitivity analysis on the effect size used in the power analysis. The required sample size for an effect size of 0.8 was determined to be 27, while for an effect size of 0.5, the required sample size increased to 67. 

## 3. Results

### 3.1. Comparison between CG and TG

Sample features and division in the CG and TG are in the following table (Table 1).

TG patients were further divided according to their medication class i.e., anticoagulants, beta-blockers, angiotensin-converting enzyme inhibitors (ACE inhibitors), or angiotensin II receptor blockers (ARBs), in the following table (Table 2).

Of the four patients who smoked more than ten cigarettes per day, three were taking anticoagulants and one beta-blocker, while of the two (<10 cigarettes per day), one was taking beta-blockers in combination with diuretics and the other ARBs.

Implant–prosthetic rehabilitation details are in the following table (Table 3).

### 3.2. Implants Survival Rate

Implant failure details and implant survival rate are in the following table (Table 4).

Implants lost during the osseointegration period were considered to have failed early, those later.

In the CG, all failed implants belonged to patients smoking more than 10 cigarettes per day.

In the TG, the rate of late implant failure was prevalent in smokers of more than ten cigarettes per day (two cases out of a total of three implants lost).

Of the patients with early failure, one was taking anticoagulants and the other beta-blockers; of those with late failure two were taking anticoagulants in combination with diuretics and the other ACE Inhibitors.

No statistically significant differences in implant survival rates (early and late failure rates) between groups were observed (*p* = 0.072). The differences between the two groups at a 95% confidence level appear not to be significant enough to reject the null hypothesis, and the two groups should be considered statistically not different.

### 3.3. Marginal Bone Loss

MBL levels recorded during the follow-up period are in the table below (Table 5).

**Figure 1:**
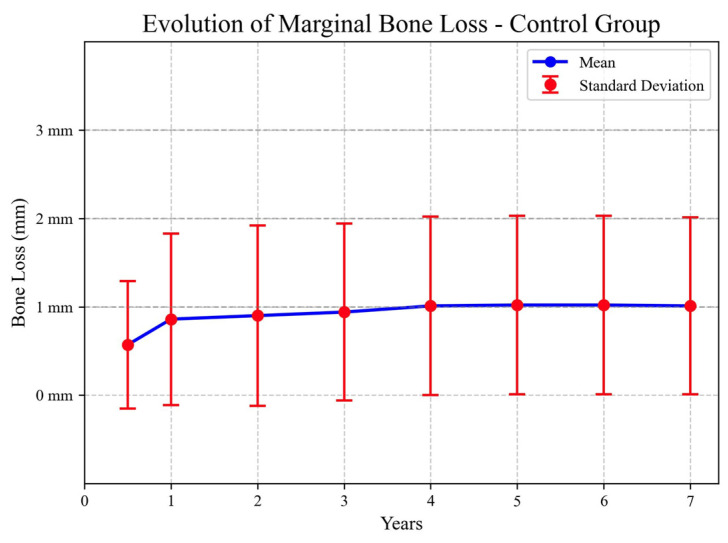
Marginal bone loss over time in CG.

The evolution of marginal bone loss over time for the CG is summarized in the following figure (Figure 1).

**Figure 2:**
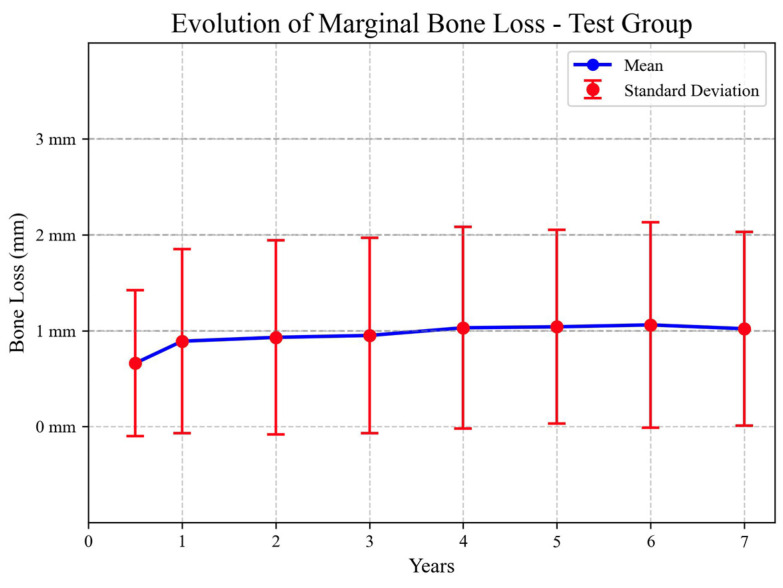
Marginal bone loss over time in TG.

The evolution of marginal bone loss over time for the TG is summarized in the following figure (Figure 2).

**Figure 3:**
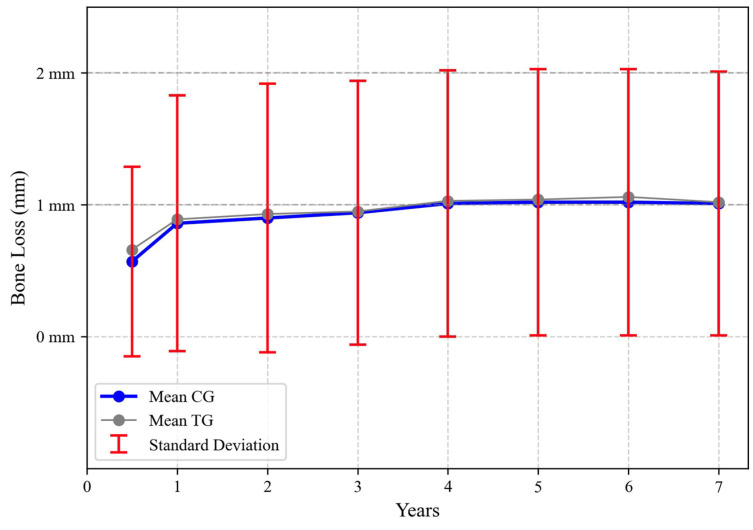
Comparison between CG and TG concerning MBL evolution.

The comparison between the CG and TG is summarized in the following figure (Figure 3).

In the CG, the average peri-implant marginal bone loss between patients who smoked less than ten cigarettes per day and non-smokers was almost similar, whereas higher values were recorded in patients smoking more than ten cigarettes per day.

In the TG, the extent of peri-implant marginal bone loss appeared quite comparable between individuals who smoked fewer than ten cigarettes daily and non-smokers. In contrast, those who smoked more than ten cigarettes per day exhibited notably greater levels of marginal bone loss around the implants. 

There was no numerical evidence of increased marginal bone loss in patients receiving a certain drug category. 

No statistically significant differences in MBL between groups were observed (*p* = 0.109). The differences between the two groups at a 95% confidence level appear not to be significant enough to reject the null hypothesis, and the two groups should be considered statistically not different.

### 3.4. Peri-Implant Parameters

Peri-implant parameters in the CG and TG are in the following table (Table 6).

In both groups BoP values were in line with PI values, suggesting a positive association between them.

The association between smoking and peri-implant parameters in the CG is reported in the following table, concerning PSR code (Periodontal screening and recording), PI + BoP (plaque index + bleeding index), and PPD (peri-implant probing depth) (Table 7).

The relation between smoking and peri-implant parameters in the TG is reported in the following table (Table 8).

Concerning peri-implant parameters, statistically significant differences were found between healthy patients and those suffering from cardiovascular diseases (0.038), showing worse peri-implant parameters at a 7-year follow-up in affected patients than in the CG; the differences between groups at a 95% confidence level were significant enough to reject the null hypothesis, and the two groups should be considered statistically different.

### 3.5. Surgical Complications

Surgical complications in the CG and TG are in the following table (Table 9).

The association between smoking and surgical complications in the CG is reported in the following table (Table 10).

The relation between smoking and surgical complications in the TG is reported in the following table (Table 11).

Except for the “bleeding” variable, the disparities in surgical complications, including edema, pain, bleeding, and wound infection, between the two groups did not reach statistical significance at a 95% confidence level, supporting the retention of the null hypothesis. The calculated *p*-value for this analysis was 0.078, further confirming the absence of statistically significant differences in surgical complications between the groups.

### 3.6. Impact of Smoking on Parameters Recorded for CG and TG

Overall, patients who smoked more than 10 cigarettes per day exhibited higher rates of implant failure, marginal bone loss, and incidence of surgical complications, as well as worse peri-implant parameters.

When assessing smoking and non-smoking patients in the whole sample, statistically significant differences were found between groups (*p* = 0.029). However, the statistical analysis of the Control Group (CG) and Test Group (TG) was infeasible due to the restricted number of smoking patients in each group, failing to meet the minimum requirements for significant inferential examination. Additionally, the association of the ‘drug factor’ in the test group could influence the obtained results, which is not available given the heterogeneity of the sample and the reduced number of patients examined. The null hypothesis could not be rejected.

### 3.7. Impact of Drugs Administered to Patients with Cardiovascular Diseases

The distribution of drugs administered in relation to peri-implant parameters and surgical complications has been summarized in the following table (Table 12).

No statistically significant differences were observed in the values of implant survival rate, marginal bone loss, peri-implant parameters, and the incidence of surgical complications concerning drug intake (*p* = 0.092). However, it is crucial to note that the statistical analysis could not be deemed eligible due to the restricted number of patients and the heterogeneity of the sample. The limited sample size and the presence of heterogeneity failed to meet the minimum requirements for a statistically significant inferential examination. Consequently, the null hypothesis could not be rejected.

### 3.8. Survival Analysis

To assess the evolution of implant survival and the risk factors over time, we have performed Kaplan–Meier and Cox hazard analysis. The results of the Kaplan–Meier analysis are visualized in Figure 1 and in the following table (Table 13).

From the values in the previous table, we can conclude that for the test group, the true survival probability at seven years is between 91% and 98%, with a confidence level of 0.95.

For the control group, the true survival probability at seven years is between 92% and 99%, with a confidence level of 0.95. 

Results for the control group are shown in Figure 2 and in the following table (Table 14).

The Cox proportional hazards model is used to investigate the relationship between survival time, implant failure, and covariates. The covariates here are anticoagulant treatments and smoking. The baseline hazard is handled non-parametrically, using Breslow’s method. In this case, the entire model is the traditional semi-parametric Cox model. Ties are handled using Efron’s method. Cox hazard model covariates and survival analysis are summarized in the following table (Table 15).

Scrutinizing the coefficients associated with the covariates, specifically the use of cardiovascular treatments and smoking, reveals an elevation in the hazard rate connected with these covariates. Notably, smoking exhibits the highest hazard ratio (92.5) and a lower standard error of the coefficient (0.91), suggesting a more precise estimate with diminished variability. The general sense that we receive from these results is that a higher risk of implant failure is associated with cardiovascular treatments and, in particular, smoking.

## 4. Discussion

The aim of this study was to conduct a comprehensive investigation and comparison of implant-related outcomes between two distinct patient groups: those who are considered healthy (CG) and those diagnosed with cardiovascular disease (TG). The primary focus was placed on assessing implant survival rates, marginal bone loss, peri-implant parameters, and surgical complications as the key outcomes of interest. The second focus was placed on the impact of smoking in both the TG and CG, as well as evaluating the influence on drugs administered to patients with cardiovascular diseases. 

The study’s findings revealed that there were no statistically significant differences in implant survival rates, both in terms of early and late failure rates, between the two patient groups. This suggests that the presence of cardiovascular disease did not have a discernible impact on the likelihood of implant failure. Additionally, there were no significant disparities concerning marginal bone loss, indicating that this crucial measure of implant stability did not significantly differ between the two groups.

Similar results were reported by Aghaloo et al. [35] and D’Ambrosio et al. [36], who, in their literature review, reported that cardiovascular diseases and related drugs do not result in a decreased rate of implant osseointegration.

Marchio et al. [37], in their clinical study, compared healthy patients and patients suffering from various systemic diseases, such as cardiovascular diseases (arrhythmia, hypertension, atrial fibrillation, bypass, and pacemaker surgery), depression, endocrine metabolic diseases (hypercholesterolemia, type II diabetes, Hashimoto’s thyroiditis), gastrointestinal diseases (gastritis, hiatal hernia, gastric ulcers), asthma, osteoporosis, and glaucoma, and did not show statistically significant differences between the groups in either implant survival rates or marginal bone loss. 

Other systemic disease-specific clinical studies have provided the same results [38,39].

However, contrary to these authors’ assertions, a possible correlation between peri-implantitis, which could affect implant success in the medium and long term, and cardiovascular pathologies might exist [40,41]. 

In contrast, when evaluating peri-implant parameters, which included PSR (Periodontal screening and recording), PI + BoP (plaque index + bleeding index), and PPD (peri-implant probing depth), the study observed statistically significant differences. Patients diagnosed with cardiovascular diseases exhibited worse peri-implant parameters during the 7-year follow-up period compared to the group of healthy individuals. This finding suggests that the presence of cardiovascular disease may be associated with a higher likelihood of suboptimal peri-implant conditions, which can impact the long-term health of dental implants.

Sumayin Ngamdu and their colleagues, in their insightful observational cross-sectional study [42], have contributed to the growing body of evidence supporting a potential connection between cardiovascular pathologies and the severity of peri-implant disease. Their research underscores the idea that cardiovascular conditions may exert an influence on the degree of peri-implant complications.

Furthermore, Wang and co-authors [43] have echoed this line of inquiry by presenting findings that demonstrate an elevated expression of pro-inflammatory cytokines [44] in patients suffering from chronic peri-implantitis and concurrently dealing with cardiovascular diseases (CVDs). This connection reinforces the significance of delving deeper into the relationship between systemic health and implant-related complications.

As for surgical complications, our study revealed a noteworthy difference between the groups in terms of bleeding, with patients affected by CVDs exhibiting a significantly higher occurrence. While some authors have postulated that this difference could be linked to the use of anticoagulant medications in the treatment of various cardiovascular diseases [20,45,46], the diversity within our sample prevented us from definitively attributing this complication to specific drugs.

About the impact of smoking on the examined groups, the data revealed a direct correlation between the number of cigarettes smoked per day and various adverse outcomes, including implant failure, marginal bone loss, peri-implant parameters, and surgical complications. This observation aligns with the findings of other researchers who have emphasized the role of smoking in issues related to both dental implants and peri-implant health [47,48,49].

However, it is worth noting that when attempting to conduct a statistical analysis comparing the CG and TG, the limited number of smokers in each group posed a challenge. The sample size of smokers in each group did not meet the minimum requirements necessary for a robust inferential analysis. This limitation underscores the need for larger and more balanced sample sizes to draw meaningful conclusions regarding the impact of smoking in the context of dental implant research.

Additionally, drugs (anticoagulants, Beta-Blockers, ACE Inhibitors, Antiplatelet Agents, ARBs, and diuretics) did not have an impact on the incidence of surgical complications, marginal bone loss, peri-implant parameters, or implant survival rate among patients with cardiovascular diseases. However, heterogeneity and a small sample size prevented a statistically meaningful inferential analysis from meeting the minimal requirements.

There is little evidence in the current literature on whether anticoagulants, Beta-Blockers, ACE Inhibitors, Antiplatelet Agents, ARBs, and diuretics may influence the stability and overall success of dental implants. The main concern is that the findings of the studies often fail to reach significance, requiring large sample sizes [50]. 

While a potential impact was not demonstrated for unselective beta-blockers like propranolol, some studies have suggested that selective beta-blockers could be correlated with better bone mineral density and other parameters related to the success of implants. [51,52,53]. 

Even though this 7-year follow-up prospective single cohort study provides insights into the long-term effects of cardiovascular diseases on dental implants, it has several limitations. The main limitation is the small sample size, especially when smokers and medications administered to patients with cardiovascular diseases were considered. This results in a limited generalizability of the study. 

Even the patient characteristics of the study may not be representative of a broader population: genetic differences, age, ethnicity, health status, and socio-economic status. 

The study setting, which is the Dental School Department of Dentistry IRCCS San Raffaele Hospital, Milan, and the specific geographic location affect the applicability of the findings to other locations.

Smoking habits and the drugs prescribed were self-reported, as they were collected through an anamnestic questionnaire. This is a limit for the study, because the patients may not accurately remember or report correctly both smoking habits and the drugs they are taking. 

The study involved patients with different cardiovascular diseases and medications. The heterogeneity in these factors may pose a challenge to making specific conclusions on the impact of each condition. Moreover, the duration and dosage of all medications were not described, so they could potentially have an impact on the outcomes.

Since the limited sample size and the presence of heterogeneity, sometimes we failed to meet the minimum requirements for a statistically significant inferential examination.

Although there are some limitations, this study is relevant for the clinician, because it adds valuable insights to the understanding of implant outcomes in patients with cardiovascular diseases receiving full-arch rehabilitation.

However, further studies are needed with longer follow-ups and larger sample sizes.

## 5. Conclusions

Within the limitations of the present study, patients with cardiovascular diseases receiving full-arch rehabilitation reported statistically significant differences from healthy subjects concerning peri-implant parameters, which were more adverse, and bleeding rates.

Further clinical studies on larger scales are required to distinguish between the examined groups due to the impact of smoking and drug intake and the possible relationship between both in the test group.

## Figures and Tables

**Table 1:** Patients’ features according to sample division in CG and TG.

	CG	TG
N° patients	26	28
Female	14	13
Males	12	15
Mean age (years)	68	69
Range age (years)	59–77	57–81
Total smokers	8	6
Smokers < 10 cigarettes/day	5	2
Smokers > 10 cigarettes/day	3	4

**Table 2:** Patient distribution according to medication category undertaken.

Drug 1	Drug 2	N° Patients
Anticoagulants		6
Beta-Blockers		7
ACE Inhibitors		5
Antiplatelet Agents		2
ARBs		2
Anticoagulants	Diuretics	3
Beta-Blockers	Diuretics	1
ACE Inhibitors	Diuretics	2

**Table 3:** Implant–prosthetic rehabilitation details according to sample division in CG and TG.

	CG	TG
Maxilla	8	9
Mandible	7	7
Both	11	12
6 axial implants	12	15
All-on-Four protocol	14	13
Total dental implants	128	142

**Table 4:** Implant failure details and implant survival rate according to sample division in CG and TG.

	CG	TG
Early failure	1	3
Late failure	2	3
Overall failure	3	6
Implant survival rate	97.66%	96.48%

**Table 5:** Mean ± standard deviation of MBL levels recorded in CG and TG.

MBL (mm)	CG	TG
6 months	0.57 ± 0.72	0.62 ± 0.76
1 year	0.86 ± 0.97	0.89 ± 0.96
2 years	0.90 ± 1.02	0.93 ± 1.01
3 years	0.94 ± 1.00	0.95 ± 1.02
4 years	1.01 ± 1.01	1.03 ± 1.05
5 years	1.02 ± 1.01	1.04 ± 1.01
6 years	1.02 ± 1.01	1.06 ± 1.07
7 years	1.01 ± 1.00	1.02 ± 1.01

**Table 6:** Peri-implant paramters in CG and TG.

	CG	TG
PSR Periodontal screening and recording		
CODE 1	12	7
CODE 2	9	9
CODE 3	4	11
CODE 4	1	1
PI (%) plaque index		
70	0	2
60	4	4
50	6	13
40	11	7
30	3	2
20	2	0
PPD (mm) peri-implant probing depth		
4	16	8
5	7	16
6	3	4

**Table 7:** Relation between number of smokers of more and less than 10 cigarettes and peri-implant parameters in CG.

	PSR CODE				PI + BoP					PPD		
	1	2	3	4	20%	30%	40%	50%	60%	4 mm	5 mm	6 mm
Smoking < 10	0	2	3	0	0	0	0	4	1	0	0	3
Smoking > 10	0	0	2	1	0	0	0	0	3	0	2	3

**Table 8:** Association between number of smokers of more and less than 10 cigarettes and peri-implant parameters in TG.

	PSR CODE				PI + BoP							PPD	
	1	2	3	4	20%	30%	40%	50%	60%	70%	4 mm	5 mm	6 mm
Smoking < 10	0	0	0	2	0	0	0	0	1	1	0	0	2
Smoking > 10	0	0	3	1	0	0	0	0	3	1	1	1	2

**Table 9:** Surgical complication in CG and TG.

	CG	TG
Edema	2	2
Post-surgical pain	3	3
Bleeding	3	8
Wound infection	1	1

**Table 10:** Relation between number of smokers of more and less than 10 cigarettes and surgical complications in CG.

	Edema	Pain	Bleeding	Wound Infection
Smoking < 10	0	0	0	0
Smoking > 10	2	1	3	1

**Table 11:** Association between number of smokers of more and less than 10 cigarettes and surgical complications.

	Edema	Pain	Bleeding	Wound Infection
Smoking < 10	0	1	2	0
Smoking > 10	2	1	4	1

**Table 12:** Distribution of drugs administered in relation to peri-implant parameters and surgical complications.

		PSR				PI + BoP					PPD			Edema	Pain	Bleeding	Wound Infection
Drug 1	Drug 2	1	2	3	4	30%	40%	50%	60%	70%	4 mm	5 mm	6 mm				
Anticoagulants		1	2	3	0	1	0	3	1	1	1	4	1	1	0	4	1
Beta-Blockers		1	3	2	1	0	1	4	2	0	2	3	2	0	0	0	0
ACE Inhibitors		2	1	2	0	1	1	2	1	0	2	2	1	0	1	4	0
Antiplatelet Agents		0	1	1	0	0	1	1	0	0	1	1	0	0	0	1	0
ARBs		1	0	1	0	0	0	1	0	1	0	1	0	0	0	0	0
Anticoagulants	Diuretics	1	2	0	0	0	1	2	0	0	1	2	0	0	1	0	0
Beta-Blockers	Diuretics	0	0	1	0	0	1	0	0	0	0	1	0	0	1	0	0
ACE Inhibitors	Diuretics	1	0	1	0	0	2	0	0	0	1	1	0	0	0	0	0

**Table 13:** Kaplan–Meier survival rates’ confidence intervals test group.

	KM Estimate—Lower Percentile (0.95)	KM Estimate—Upper Percentile (0.95)
6 months	0.9448	0.9964
3 years	0.9359	0.9931
5 years	0.9174	0.9851
7 years	0.9174	0.9851

**Table 14:** Kaplan–Meier survival rates’ confidence intervals control group.

	KM Estimate—Lower Percentile (0.95)	KM Estimate—Upper Percentile (0.95)
6 months	0.9458	0.9988
3 years	0.9389	0.9960
5 years	0.9291	0.9923
7 years	0.9291	0.9923

**Table 15:** Summary of Cox hazard model covariates and survival analysis.

	Coefficient	exp(coef.)	se(coef)	z	*p*	−log2(p)
Group	−0.14	0.87	0.94	−0.15	0.88	0.18
Covariate 1 (anticoagulant treatment)	2.90	18.20	1.29	2.25	0.02	5.36
Covariate 2 (smoking)	4.53	92.52	0.91	5.00	<0.0005	20.72

## Data Availability

The original contributions presented in the study are included in the article, further inquiries can be directed to the corresponding author.
